# Parasternal intercostal muscle ultrasound in chronic obstructive pulmonary disease correlates with spirometric severity

**DOI:** 10.1038/s41598-018-33666-7

**Published:** 2018-10-15

**Authors:** Peter Wallbridge, Selina M. Parry, Sourav Das, Candice Law, Gary Hammerschlag, Louis Irving, Mark Hew, Daniel Steinfort

**Affiliations:** 1 Department of Respiratory and Sleep Medicine, Royal Melbourne Hospital, Parkville, Australia; 2 Department of Medicine, Royal Melbourne Hospital, The University of Melbourne, Parkville, Australia; 3 Department of Physiotherapy, The University of Melbourne, Parkville, Australia; 4 Melbourne EpiCentre, Melbourne Health and The University of Melbourne, Parkville, Australia; 5 Department of Radiology, Royal Melbourne Hospital, Parkville, Australia; 6 Public Health and Preventative Medicine, Monash University, Clayton, Australia

## Abstract

In chronic obstructive pulmonary disease (COPD), loss of computed tomography (CT)-measured intercostal mass correlates with spirometric severity. Intercostal muscle ultrasound offers a repeatable and radiation-free alternative, however requires validation. We aimed to determine the reliability of parasternal intercostal muscle ultrasound, and the concurrent validity of parasternal ultrasound with clinicometric parameters. Twenty stable COPD patients underwent ultrasound measurement of thickness and echogenicity of 2^nd^ and 3^rd^ parasternal intercostal muscles, dominant pectoralis major and quadriceps, and diaphragm thickness; spirometry; and chest CT. Intra-rater intraclass correlation (ICC) for ultrasound intercostal thickness was 0.87–0.97 depending on site, with echogenicity ICC 0.63–0.91. Inter-rater ICC was fair to excellent. Ultrasound intercostal thickness moderately correlated with FEV_1_% predicted (r = 0.33) and quadriceps thickness (r = 0.31). Echogenicity correlated negatively with FEV_1_% predicted (r = −0.32). CT-measured lateral intercostal mass correlate negatively with parasternal ultrasound intercostal thickness. These data confirm ultrasound of parasternal intercostal musculature is reproducible. Lower intercostal muscle quantity and quality reflects greater COPD spirometric severity. This novel tool may have biomarker potential for both the systemic effects of COPD on muscle as well as local disruption of respiratory mechanics. The negative correlation between CT and ultrasound measurements may reflect complex site-dependent interactions between respiratory muscles and the chest wall.

## Introduction

Chronic obstructive pulmonary disease (COPD) is characterised by non-reversible airflow obstruction with persistent respiratory symptoms^1^ and associated extra-pulmonary effects of weight loss and muscle wasting, reflecting its systemic impact. In COPD, impaired muscle strength and composition (muscle quality) is associated with poorer clinical outcomes^2,3^ and increased healthcare utilization^4^.

Bedside ultrasonography enables evaluation of muscle quantity and composition^5–7^, with increasing muscle echogenicity (a measure of muscle quality, with higher scores indicating poorer muscle quality) associated with increased muscle fat infiltration in healthy individuals^8^ as well as in critical illness^9^. Dynamic changes in muscle cross-sectional area and echogenicity correlate with strength and function during critical illness^10^.

In COPD, ultrasound quadriceps bulk is reduced compared to controls^11^, and predicts readmissions and mortality^12^. Increased echogenicity is associated with higher spirometry-defined COPD severity^13^, and low muscle attenuation on computed tomography (CT) (reflecting poorer quality muscle), is more common than in control subjects^14^.

Changes in chest wall musculature may reflect combined systemic effects and local disruption of respiratory mechanics due to airflow obstruction and hyperinflation in COPD. Imaging of thoracic musculature may therefore be more instructive than peripheral musculature. Animal models have established the impact of increasing lung volume on the ability of intercostal muscles to alter airway opening pressure in addition to a reduction in the ability of the diaphragm to generate force^15^. It has been shown that a lower mass of intercostal muscles measured by CT correlates with greater COPD spirometric severity^16^, and exacerbation frequency^17^. However CT assessment of intercostal muscles is resource intensive, generally requires specialized expertise, exposes patients to ionizing radiation, and cannot be repeated frequently.

Ultrasound overcomes many of these drawbacks given its lack of ionising radiation, portability, and ability to examine other relevant muscle groups including the diaphragm and quadriceps. Chest ultrasound is already in routine clinical use for the investigation and management of pleural and parenchymal lung disease^18–22^. In healthy volunteers, ultrasound has been used to study intercostal muscles in the easily-accessible anterior parasternal region, but is yet to be applied to patients with COPD^23,24^.

In this pioneering study, we aimed to demonstrate feasibility and reproducibility of sonographic measurement of parasternal intercostal muscles in patients with COPD. We hypothesised that ultrasound measurement of parasternal intercostal muscles would be feasible in patients with COPD and have high intra- and inter-rater reliability. We also hypothesised that ultrasound intercostal muscle quantity measured by thickness, and quality (measured by grayscale analysis), would reflect spirometric COPD severity.

## Methods

Ultrasound examination was performed on 20 patients with stable COPD recruited from the respiratory clinic of a university-affiliated tertiary referral hospital in Melbourne, Australia. We included patients aged >18 years with COPD confirmed by spirometry (post-bronchodilator forced expiratory ratio <70%)^1^. Participants were required to have undergone chest CT within 6 months of enrolment. We excluded patients with an exacerbation of COPD requiring antibiotic or oral corticosteroid therapy within previous 6 months, or inability to provide informed consent. Baseline demographic data, short-form international physical activity questionnaire (IPAQ or IPAQ-Elderly for patients aged ≥65 years) and Charlson comorbidity index were obtained. Informed consent was obtained from all participants, and this project and its’ protocol received ethics approval by the Melbourne Health Human Research Ethics Committee (LNR/15/MH/405) and was carried out in accordance with local and national guidelines and regulations. The reporting of this study was in line with COnsus-based Standards for the selection of health Measurement INstruments (COSMIN) guidelines^25^.

### Ultrasound examination

Ultrasound was performed by a single operator (PW) with 8 years of ultrasound experience and qualifications in respiratory ultrasound. B-mode images were obtained utilising a 6–14 mHz linear array on a MindRay TE-5 machine (Shenzhen Mindray Bio-Medical Electronics Co. Ltd. Shenzen, China) with the participant at 45 degrees. Depth and gain were standardised using the same image presets between patients.

**Figure 1:**
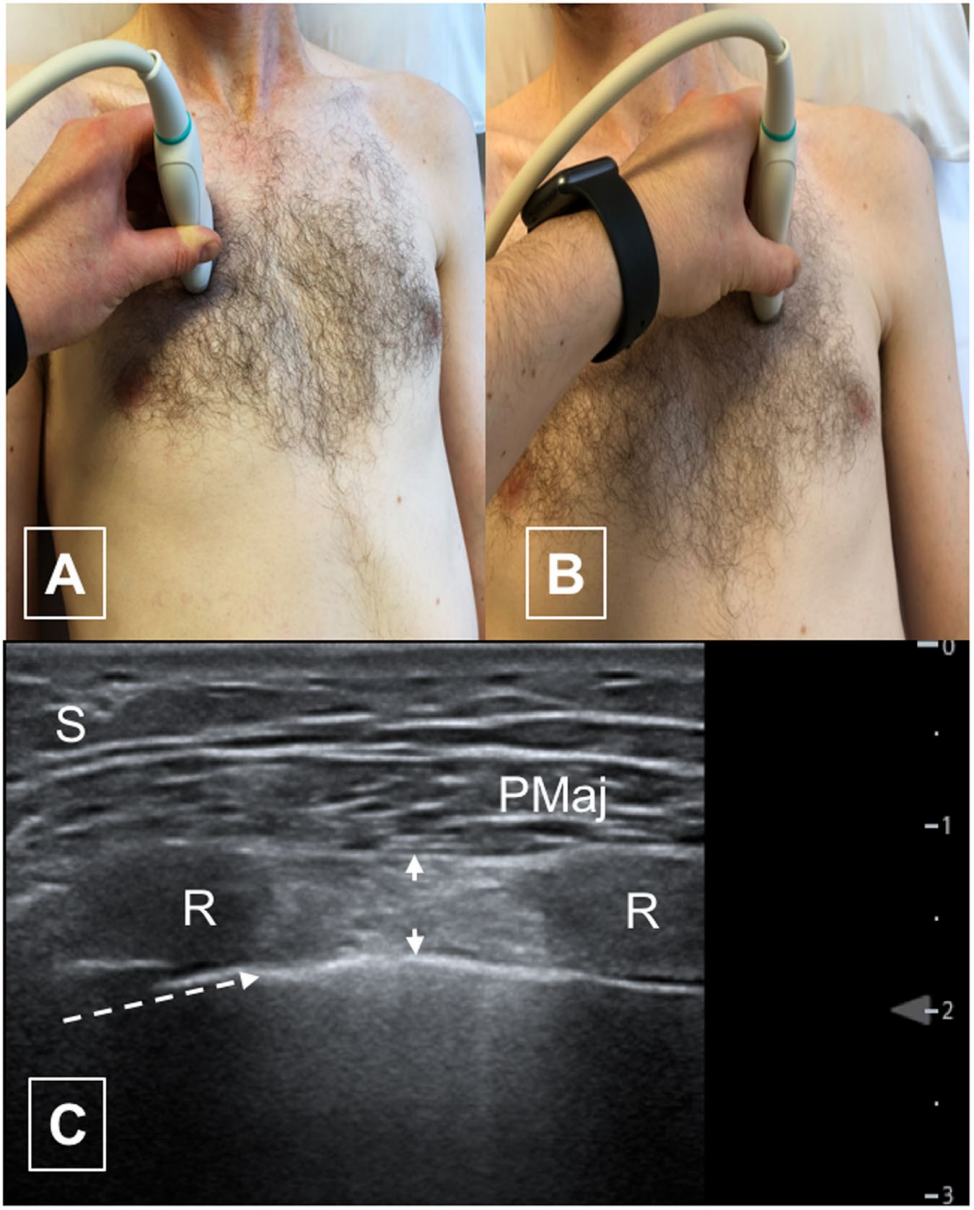
Parasternal intercostal ultrasound. (**A**) Position of probe for right second intercostal space. (**B**) Position of probe for left second intercostal space. (**C**) Typical ultrasound image of parasternal intercostal muscle (left 2^nd^ intercostal space. S – subcutaneous fat PM – pectoralis major muscle. R – rib Arrow heads – outline of intercostal muscle. Dotted arrow – pleural line.

**Figure 2:**
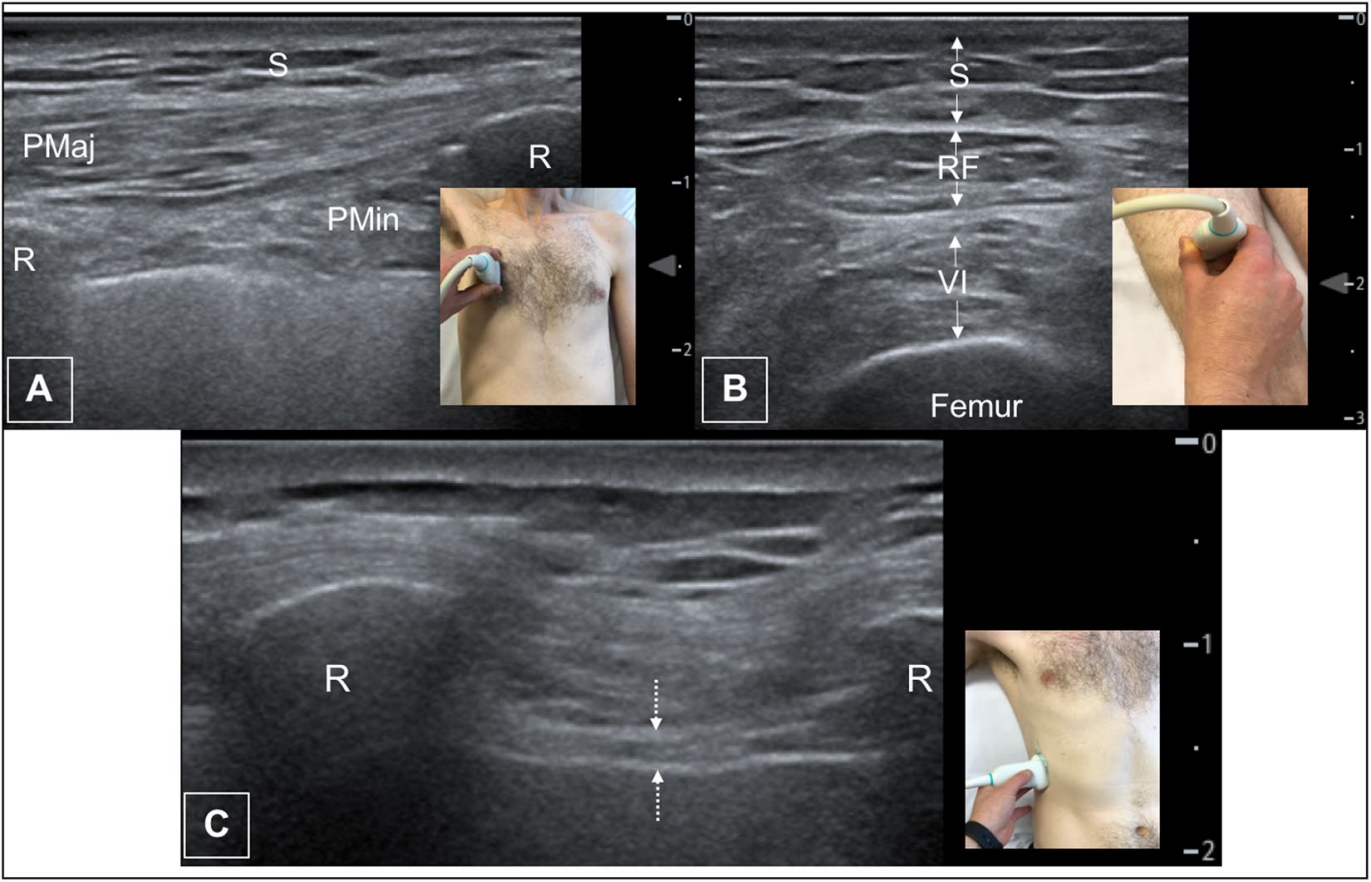
Pectoralis major, femoral muscle and diaphragm ultrasound images. (**A**) Typical ultrasound image of pectoralis major. (**B**) Typical ultrasound image and probe position of femoral muscles. (**C**) Typical ultrasound image and probe position of diaphragm thickness. S – subcutaneous fat PMaj – pectoralis major. PMin – pectoralis minor R – rib RF – rectus femoris VI – vastus intermedius. Dotted arrow – diaphragmatic surface.

Still images were taken of the 2^nd^ and 3^rd^ parasternal intercostal muscles bilaterally in the sagittal plane at end-tidal inspiration with a window visualising the 2^nd^/3^rd^, and 3^rd^/4^th^ ribs respectively (Fig. 1); dominant pectoralis major muscle (sagittal plane at the mid-point of the muscle), diaphragm thickness (end-tidal expiration in the right anterior axillary line between the 8^th^ and 9^th^ ribs in the coronal plane)^26^, and quadriceps (vastus intermedius and rectus femoris thickness and echogenicity) approximately 15 cm above the superior border of the patella. (Fig. 2) All sites were imaged twice to allow for assessment of intra-operator reliability, with the probe removed from the patient between measurements with less than 1 minute between scans and no change in patient condition or positioning. Subcutaneous fat thickness was measured at all sites.

**Figure 3:**
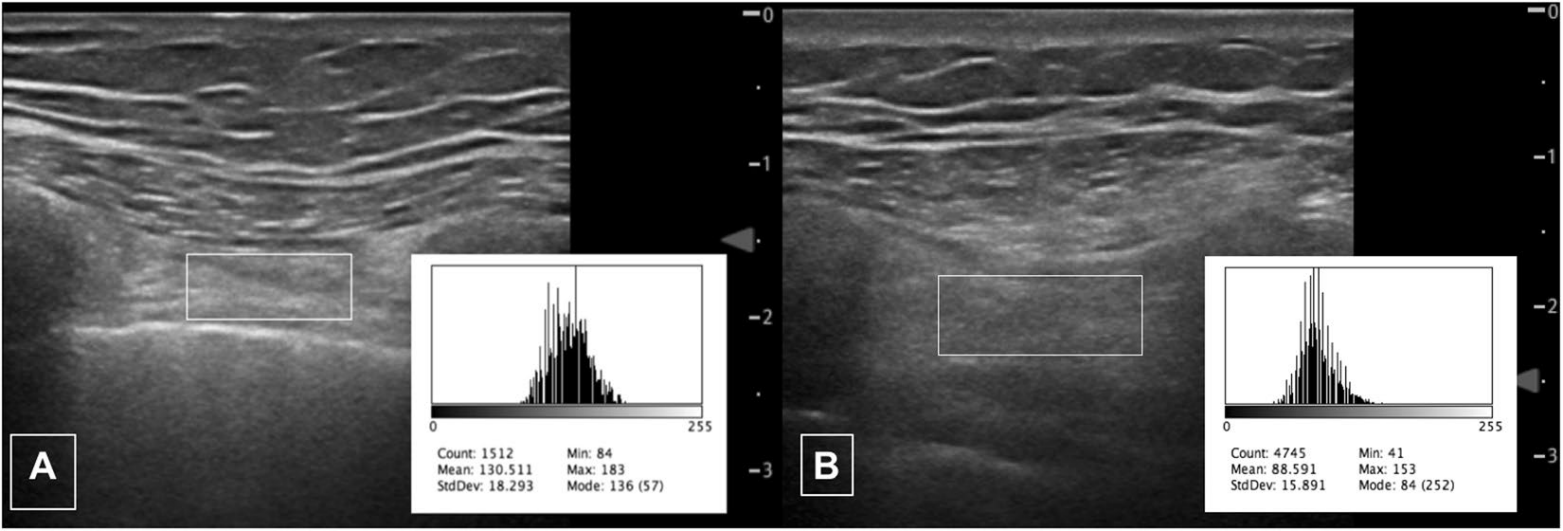
Echogenicity of parasternal intercostal muscle. Typical ultrasound images of parasternal intercostal muscle (right 3^rd^ intercostal muscle) with square to measure region of interest for quantitative grayscale analysis showing (**A**) higher echogenicity and (**B**) lower echogenicity. Insets depict respective histograms of grayscale analysis (range 0 [black] to 255 [white]) with mean value recorded.

Measurements were performed on stored images using freely available ImageJ software (https://imagej.nih.gov/ij/index.html NIH, Bethesda, MD, USA). Muscle thickness in millimetres was between the inner and outermost echogenic layer of the muscle fascial borders. Muscle echogenicity was calculated utilising the square method^27^ to define the region of interest for analysis using ImageJ histogram function (Fig. 3). Images were reviewed by a second reader (GH) with respiratory ultrasound experience to assess inter-rater reliability.

**Figure 4:**
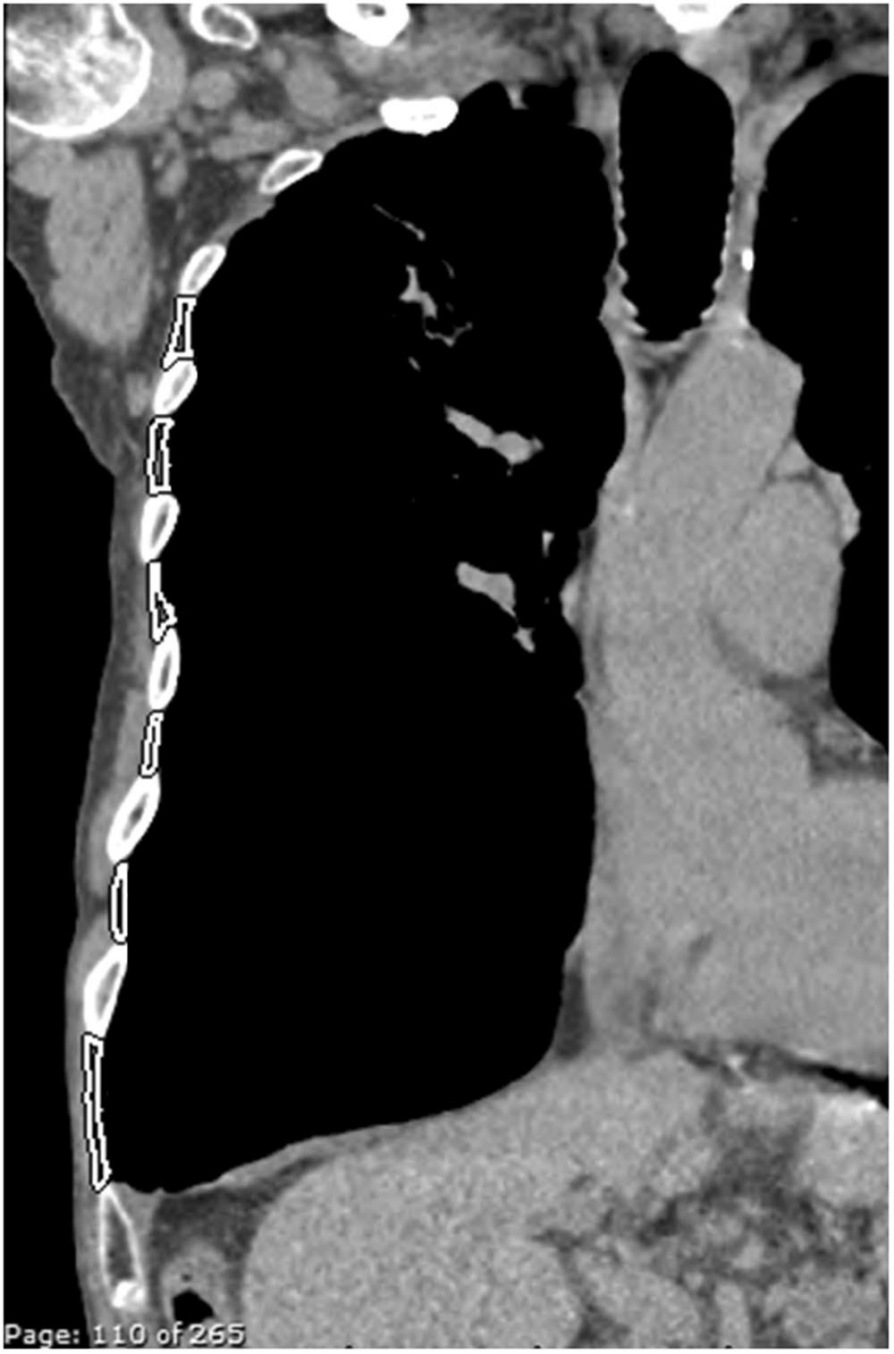
CT image of lateral chest wall internal intercostal muscles. Coronal image from chest CT at lateral arc of 1^st^ rib with ROI surrounding boundaries of 3^rd^–8^th^ internal intercostal muscles.

Images from CTs were reviewed by a chest radiologist (CL) blinded to ultrasound results, who identified the outline of the third to eighth internal intercostal muscles in the plane of the lateral arc of the first rib as previously described by Park^16^ (Fig. 4). Within this region of interest, muscle area was recorded utilising a Hounsfield unit threshold of −29 to +150HU^28^. Both total CT-intercostal muscle area and ‘CT intercostal index’ (total CT intercostal area/body-mass-index (BMI)) were calculated. Results were collated on electronic spreadsheet (Microsoft Excel, Microsoft Corporation).

Correlations between ultrasound, CT measurements, COPD severity (FEV_1_ and FEV_1_% predicted) and patient factors (BMI and Charlson comorbidity index) were sought.

### Statistical analysis

Demographic data were analysed using GraphPad Prism 7 (GraphPad Software Inc., CA USA), and correlation analysis utilising R Package (R Foundation for Statistical Computing, Vienna, Austria)^29–31^. Correlation was measured using Pearson correlation coefficient with intra- and inter-rater reliability analysed using intraclass correlation coefficient. P-values were not calculated for these data given the limited number of subjects and exploratory nature of analyses, apart from partial correlation analysis^32^ for intercostal ultrasound measurements adjusting for repeated single-operator and between measurements with analysis performed using SPSS 24 (IBM Corp. 2016. IBM SPSS Statistics for Windows, Version 24.0. Armonk, NY: IBM Corp.).

Data are described by median and interquartile range unless otherwise specified.

## Results

Twenty patients underwent ultrasound examinations. Four patients were excluded from CT correlation analysis due to inability to format CT images. Ultrasound data from these patients are included in reliability and correlation analyses. Participants typically reported low (52%) or moderate (32%) levels of physical activity on IPAQ. Demographic data are recorded in Table 1. Ultrasound findings are recorded in Table 2.

**Table 1:** All data are median (IQR)

Demographics	n = 20
Sex	Male: 16 (80%) Female: 4 (20%)
Age	71.5 (62.3–78.8)
BMI	23.5 (20.9–30)
Current smoker	Yes: 4 (20%) No: 16 (80%)
FEV_1_ (litres)	1.15 (0.99–1.81)
FEV_1_ (% predicted)	45 (34–74)
Diffusion capacity (% predicted)	44 (34–57)
6-minute walk test distance (metres) (n = 14)	320 (253–455)
Age-adjusted Charlson Comorbidity Index	5 (4–5)

.

**Table 2:** Ultrasound Findings.

Site	Thickness (mm) Median (IQR)	Echo intensity Median (IQR)	Subcutaneous fat thickness (mm) Median (IQR)
Pectoralis major	6.4 (5.7–9.6)	100.2 (81.5–110.3)	6.9 (3.4–9.3)
Left 2^nd^ intercostal	4.2 (3.3–5.4)	110 (96.2–117.5)	3.9 (2.5–6.1)
Left 3^rd^ intercostal	5.3 (3.2–7.0)	105 (86.2–120.4)	4.8 (3.2–7.9)
Right 2^nd^ intercostal	4.1 (2.9–5.9)	105.5 (86.5–113.3)	4.9 (2.9–7.7)
Right 3^rd^ intercostal	6.0 (3.9–7.5)	96.2 (82.2–115.2)	6.1 (3.7–8.9)
Rectus femoris	5.9 (4.6–7.7)	93.6 (88.1–106.2)	5.9 (3.1–7.8)
Vastus intermedius	7.3 (5–8.6)	93.6 (66.8–113.6)	5.9 (3.1–7.8)
Diaphragm	1.97 (1.59–2.25)	N/A	N/A

IQR – interquartile range.

### Intercostal ultrasound

Parasternal intercostal muscles were visualised in all patients, with difficulty in one patient with a pes excavatum deformity. Median intercostal thickness (interquartile range) were 4.2 mm (3.3–5.4) and 5.3 mm (4.5–7.0) for left second and third parasternal intercostals respectively and 4.1 mm (2.9–5.9) and 6 mm (3.9–7.5) for second and third right intercostals. (Table 2, Fig. 1) Echogenicity varied between sites, with a wide range of values (47–151). (Table 2) When analysing echogenicity data, the impact of acoustic shadowing due to ribs was apparent (Fig. 2).

Partial correlation analysis was performed to assess for linear relationships between echogenicity, intercostal muscle thickness and density at different sites adjusting for reviewers and two measurements per patient. The correlation for muscle thickness between sites ranged from 0.551 to 0.627 (p < 0.001 for all) with density ranging from −0.261 to −0.348 (p 0.022 to 0.002). Partial correlation for echogenicity between sites ranged from 0.276 to 0.444 (p 0.015 to <0.001).

### Parasternal intercostal ultrasound and airflow obstruction

**Figure 5:**
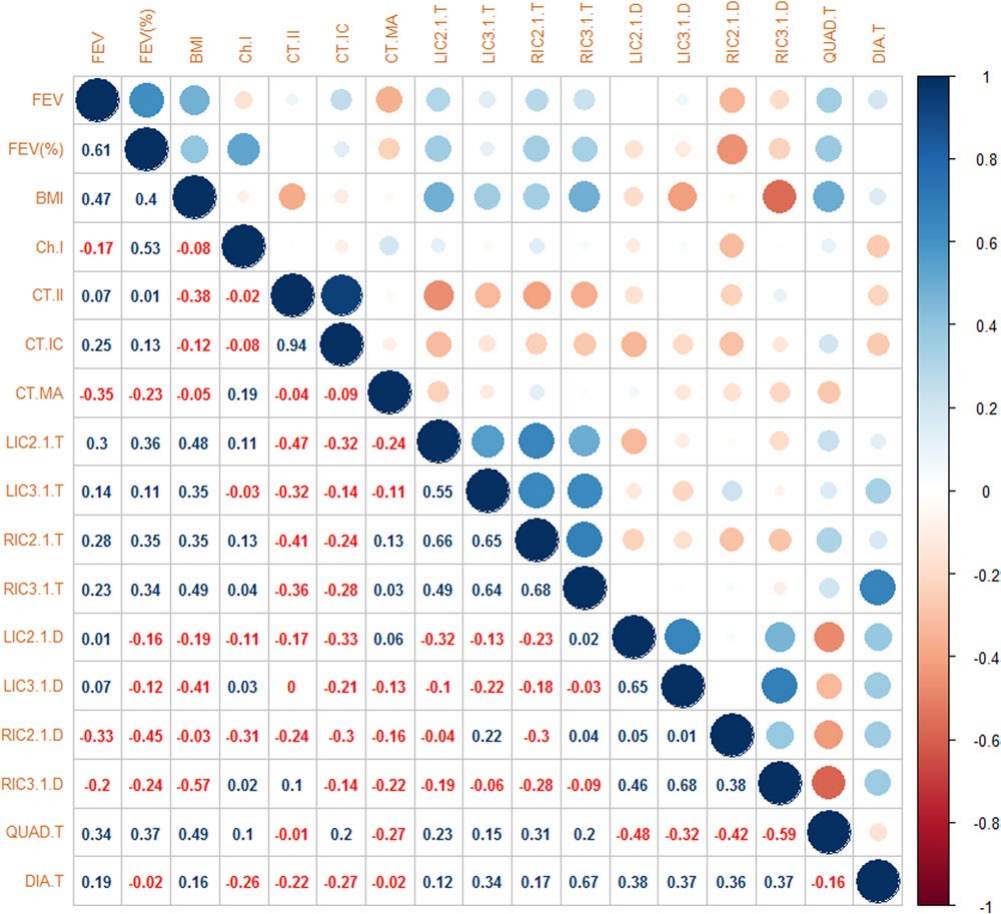
Correlation matrix. BMI – body mass index Ch.I – Charlson comorbidity index CT.II – CT intercostal index CT.IC – Total CT intercostal muscle area CT.MA – CT intercostal muscle mean attenuation LIC2.1.T – 2^nd^ left intercostal thickness LIC3.1.T – 3^rd^ left intercostal thickness. RIC2.1.T – 2^nd^ right intercostal thickness RIC3.1.T – 3^rd^ right intercostal thickness. LIC2.1.D – 2^nd^ left intercostal density LIC3.1.D – 3^rd^ left intercostal density RIC2.1.D – 2^nd^ right intercostal density RIC3.1.D – 3^rd^ right intercostal density Quad.T – rectus femoris thickness Dia.T – diaphragm thickness.

Assessment of linear relationships between intercostal ultrasound measurements and COPD-related disease markers are summarised in Fig. 5. Multiple linear regression analysis was used to estimate the variation in FEV_1_% with intercostal muscle thickness and density as explanatory factors. Utilising this model, mean intercostal thickness had a moderate positive correlation with %predicted FEV_1_, with r = 0.33. R-values varied between sites, weakest at the left third intercostal space (r = 0.11) and strongest at the left second intercostal (r = 0.36) (Fig. 5). The direction of this relationship supports our hypothesis that greater parasternal intercostal thickness would be associated with better lung function.

Ultrasound-measured mean echogenicity showed a moderate negative correlation with FEV_1_% predicted, (r = −0.32) on regression modelling. Higher echogenicity scores reflect reduced muscle quality, and given CT data that intercostal attenuation correlates with FEV_1_, we had hypothesised that echogenicity would correlate negatively. Values for independent sites however varied widely which likely reflects the impact of adjacent ribs within the narrow intercostal space, with ultrasound scatter and reflection previously being suggested to account for the majority attenuation caused by bone^33^, which could plausibly explain echogenicity variation.

### Ultrasound muscle relationships

Intercostal thickness was weak-to-moderately positively correlated to quadriceps thickness (r = 0.15–0.31), with a weak-to-moderate positive correlation to diaphragm thickness (r = 0.12–0.67) (Fig. 5). The degree of correlation between FEV_1_% predicted and quadriceps thickness approximated that seen in the second intercostal spaces (r = 0.37). The similar strength of correlation is reassuring, particularly given the strong relationship between quadriceps size and clinically meaningful outcomes in COPD^2,12^.

### CT relationships

CT-measured intercostal mass only weakly correlated with FEV_1_% predicted (r = 0.13) and had a moderate negative correlation with parasternal intercostal thickness, ranging between r = −0.32 to −0.14). Additionally, CT-measured intercostal mass negatively correlated with diaphragm thickness (r = −0.27). CT-measured intercostal density had a weak negative correlation with ultrasound measured intercostal echogenicity.

### Reliability

Intraclass correlation (ICC) for repeated measures (intra-rater reliability) of intercostal muscles depended on site, ranging between 0.87–0.97 for thickness, and 0.0.63–0.91 for echogenicity (Table 3). This confirms the high intra-rater reliability of ultrasound measurements of parasternal intercostal muscles. Intercostal inter-rater reliability was also fair to excellent with ICC between 0.601–0.803 for thickness and 0.685–0.841 for echogenicity.

**Table 3:** Intraclass correlation – intra-observer variation.

	Intra-Reviewer 1		Intra-Reviewer 2		Inter-reviewer	
	r	Confidence interval	r	Confidence Interval	r	Confidence interval
RIC2.THICK	0.931	(0.837–0.972)	0.894	(0.757–0.957)	0.601	(0.392–0.789)
RIC3.THICK	0.965	(0.916–0.986)	0.773	(0.519–0.903)	0.682	(0.493–0.839)
LIC2.THICK	0.871	(0.707–0.945)	0.884	(0.734–0.952)	0.803	(0.661–0.905)
LIC3.THICK	0.970	(0.928–0.988)	0.919	(0.810–0.967)	0.668	(0.474–0.830)
RIC2.DEN	0.812	(0.589–0.920)	0.812	(0.591–0.921)	0.685	(0.496–0.840)
RIC3.DEN	0.627	(0.274–0.832)	0.739	(0.457–0.887)	0.722	(0.545–0.861)
LIC2.DEN	0.868	(0.702–0.945)	0.811	(0.588–0.920)	0.817	(0.682–0.913)
LIC3.DEN	0.908	(0.785–0.962)	0.903	(0.771–0.961)	0.841	(0.717–0.927)

Intra-reviewer 1: ICC of reviewer 1 over two measurements.

Intra-reviewer 2: ICC of reviewer 2 over two measurements.

Inter-reviewer: ICC of all measurements for both reviewers by site.

RIC2/3.THICK – 2^nd^/3rd right intercostal thickness measurements.

LIC2/3.THICK – 2^nd^/3^rd^ left intercostal thickness measurements.

RIC2/3.DEN – 2^nd^/3^rd^ right intercostal echogenicity measurements.

LIC2/3.DEN – 2^nd^/3^rd^ left intercostal echogenicity measurement.

The reliability of this technique appears to be reduced by obesity, with lower ICC for thickness (0.227–0.577) in obese subjects (BMI >30), particularly in the 3^rd^ intercostal space; the increased subcutaneous fat thickness in this space may play a role. The ICC for density measurements is also reduced (0.547–0.927). It is worth noting that the confidence intervals are wide due to the small number (n = 5) of obese patients.

## Discussion

In this pilot study, ultrasound measurement of parasternal intercostal muscles in COPD was feasible and reproducible. Importantly, changes in muscle quantity and quality reflected spirometric disease severity. This novel and accessible intercostal muscle assessment method in COPD can be easily repeated, and has potential biomarker utility in longitudinal observational and interventional COPD studies.

The relationships found between ultrasound measures of intercostal muscle thickness, echogenicity and FEV_1_% predicted supported our hypotheses. The strength of the positive correlation between intercostal muscle thickness and FEV_1_ was like that between quadriceps thickness and FEV_1_% predicted. Furthermore, our findings of a moderate correlation between quadriceps thickness and FEV_1_% predicted is similar to other authors^13^ even though the site of measurement differed.

We found that higher muscle echogenicity (indicating poorer muscle quality) negatively correlated with COPD severity. Previous histological studies have shown that parasternal intercostal muscles undergo remodelling in COPD^34^, which is thought to reflect muscle recruitment. To our knowledge there are no data looking at imaging correlates of these changes, although increased muscle echogenicity on ultrasound is associated with increased lipid content on biopsy^5,6^. Park and colleagues have previously demonstrated that lower lateral intercostal muscle CT-measured attenuation (reflecting muscle fat deposition) is associated with lower FEV_1_^16^.

Intraclass correlation for thickness measurements ranged between 0.87–0.97 for a single reader, suggesting excellent repeatability. The measures of parasternal muscle echogenicity were less reliable, with ICC between 0.63 and 0.98; we postulate that this is due to the impact of acoustic shadowing from adjacent ribs. We attempted to minimise this by using the square rather than trace method for determining the region of interest for histogram analysis. The inter-rater reliability was not as strong for thickness, particularly in the third intercostal spaces bilaterally. This is postulated to be due to two factors, firstly related to difficulties reviewing thin muscles (typically 4–5 mm) from stored images given ultrasound is a ‘real-time’ imaging technique; and secondarily because the trangularis sterni muscle is in intercostal spaces caudal to the second space and may have been inadvertently included in parasternal intercostal measurements within the third intercostal space.

When evaluating the CT data, there was a negative correlation between ultrasound-measured parasternal intercostal thickness and CT-defined intercostal mass, as well as CT- and US-measured echogenicity. The negative correlation between the CT and ultrasound measures of density are as expected, as lower density on CT reflects increased fat infiltration and therefore poorer muscle quality, whereas the opposite is true with ultrasound echogenicity, with increased values representing poorer quality muscles.

In contrast, the negative correlation between CT-measured intercostal mass and ultrasound-defined intercostal thickness was surprising. It is worth noting that the action (expiration or inspiration) of internal intercostal muscles change as a function of their positioning within the chest wall^35^, with parasternal intercostals acting purely on inspiration, and internal intercostals elsewhere in the chest wall exhibiting an expiratory effect. Additionally, there is a gradient of mechanical advantage that decreases moving from caudal to cranial, and rostral to ventral. We postulate that the relationship between intercostal muscle size may reflect different muscle roles and recruitment in patients with COPD dependent on chest wall site whereas global muscle quality is similar throughout the intercostal muscles, regardless of function. Alternately, a more complex non-linear relationship may be present, with definitive conclusions unable to be drawn from our small sample. Either of these hypotheses require validation in larger populations (including control subjects) to further elucidate this relationship.

It is worth noting that these results are from scans performed by an experienced single operator, and therefore the reproducibility of these results remains to be established. We believe that this technique is relatively simple when compared to other commonly used musculoskeletal ultrasound measurements such diaphragm ultrasound, and therefore anticipate that parasternal ultrasound will be easily implemented, although this remains to be established. We plan to study this in future work.

### Future directions

Given the reliability of ultrasound measurement and the direction of relationships as expected, we believe that there are benefits in this approach over the CT method. Firstly, the lack of ionising radiation is important, particularly given these patients are increasingly likely to undergo CT scanning for other indications such as lung cancer screening^36^. Ultrasound is readily repeatable, opening up new opportunities to assess function and response to therapies in patients with COPD, and given the benefits of parenchymal ultrasound in patients with acute respiratory failure^22^, the ready applicability and ability to be added to current imaging protocols shows promise.

It is remains to be seen whether there are differences between stable and exacerbating populations, and whether intercostal muscles can undergo dynamic changes described in other muscle groups^10^. The use of controls would also allow delineation of the role of hyperinflation, although it is worth noting that data in healthy individuals did not find thoracic-volume dependent changes in intercostal thickness or intercostal space distance in the second interspace bilaterally^23^. This would support data that the effect of hyperinflation on inspiratory muscles is largely due to alterations in direction of rib displacement^15^, with reduction in change in airway opening pressure as intrathoracic pressure increases. We have presented data that therapies aimed at reducing hyperinflation result in changes in intercostal muscles^37^.

The data surrounding quadriceps muscle quantity and quality in COPD demonstrate the clinical utility of lower limb ultrasound^2–4,11,12^, and given intercostal bulk is also reduced in COPD but has a more direct impact on respiratory mechanics the potential role of this biomarker is wide. We believe that a study comparing quadriceps and intercostal ultrasound in patients with COPD, focussing on patient-specific outcomes such as exercise capacity and exacerbations would provide valuable data further separating the contributions of systemic and respiratory muscle quantity and quality changes.

Finally, as previously mentioned, the reproducibility of this technique is yet to be established, and the reliability of parasternal intercostal ultrasound with different levels of operator experience requires study.

## Conclusion

In stable COPD, ultrasound measurements of parasternal intercostal muscle thickness and density (quality) correlate more strongly with severity of airflow obstruction as measured by FEV_1_ than CT-measured muscle mass, and this measurement is obtained without the need for ionising radiation. Reduced muscle quality (as measured by echogenicity) is correlated with muscle thickness. Ultrasound measurements are reproducible, with good to excellent intra-rater reliability and fair to excellent inter-rater reliability. Our data suggest US assessment of respiratory musculature may provide useful information in clinical assessment of COPD, offering a promising, reliable, and repeatable biomarker for muscle assessment without the risk of ionising radiation. Further study in larger cohorts of patients including controls are required to further delineate these relationships.

## Data Availability

The datasets generated during and/or analysed during this study are available from the corresponding author on reasonable request.
